# Controllable Valley Polarization Using Silicene Double Line Defects Due to Rashba Spin-Orbit Coupling

**DOI:** 10.1186/s11671-019-3196-3

**Published:** 2019-11-27

**Authors:** Hongyu Tian, ChongDan Ren, Benhu Zhou, Shaoyin Zhang, Weitao Lu, Yunfang Li, Jing Liu

**Affiliations:** 1 Department of Physics, Zunyi Normal University, Kweichow, 563002 China; 2 Department of Physics, Shaoyang University, Shaoyang, 422001 China; 3 School of Physics and Electronic Engineering, Linyi University, Linyi, 276005 China; 4 School of Mechanical and Vehicle Engineering, Linyi University, Linyi, 276005 China; 5 Linyi University, Linyi, 276005 China

**Keywords:** Silicene, Line defect, Rashba spin orbit coupling, Valley polarization

## Abstract

We theoretically investigate the valley polarization in silicene with two parallel line defects due to Rashba spin-orbit coupling (RSOC). It is found that as long as RSOC exceeds the intrinsic spin-orbit coupling (SOC), the transmission coefficients of the two valleys oscillate with the same periodicity and intensity, which consists of wide transmission peaks and zero-transmission plateaus. However, in the presence of a perpendicular electric field, the oscillation periodicity of the first valley increases, whereas that of the second valley shortens, generating the corresponding wide peak-zero plateau regions, where perfect valley polarization can be achieved. Moreover, the valley polarizability can be changed from 1 to −1 by controlling the strength of the electric field. Our findings establish a different route for generating valley-polarized current by purely electrical means and open the door for interesting applications of semiconductor valleytronics.

## Introduction

Silicene, a low-buckled monolayer-honeycomb lattice of silicon atoms, is a potentially attractive alternative to graphene for valleytronic applications. The low-buckled structure gives rise to relatively large spin-orbit coupling (SOC) in silicene, and a sizable energy gap of approximately 1.55 meV is estimated at the Dirac points *K* and *K*^′^[1] Different from graphene, the low energy dispersion relation of silicene is parabolic rather than linear form. Facilitated by the buckling structure, the band structure of silicene can be controlled by applying an electric field, and even a topological phase transition from a quantum spin Hall insulator to a quantum Valley Hall insulator may occur[2, 3]. Silicene has been successfully synthesized on the surface of substrates such as Ag(111), Ir(111), and ZrB2(0001)[4–6], and its free-standing stable structure has also been predicted in several theoretical studies[7]. Most importantly, a room-temperature silicene field-effect transistor (FET) has been successfully observed experimentally[8]. The electric field tunability and compatibility with existing silicon-based devices make silicene a potential two-dimensional material for application in next-generation valleytronics.

**Fig. 1:**
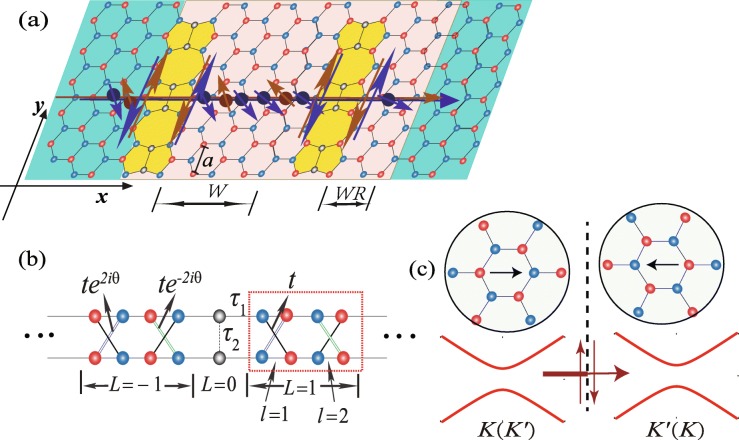
**a** Schematic diagram of the precession process of the states (*K*,*↑*)(red sphere) and (*K*^′^,*↓*)(blue sphere) through a silicene sheet with two parallel line defects, where the blue (red) circle denotes the *A*(*B*) sublattice. The states (*K*,*↑*) and (*K*^′^,*↓*) circulate along the pseudo-edge, and the RSOC as well as electric field is assumed to exist on the french grey region. *W*(*W*=2) and *WR*(*WR*=1) represent the width of the scattering region in units of $\sqrt {3}a$. **b** The simplifed lattice model of the infnite silicene with a line defect, where *θ*=*k*_*y*_*a* and the dotted rectangle corresponds to a supercell. In the unit cell, the lattice points are specified by a set of indices (*L,l*). **c** The transmission for one spin state in the *K*(*K*^′^) valley across line defect with inversion domain boundaries. The insets show the orientation of the crystalline lattice in the two domains separated by the line defect (dashed line). The thick/thin lines indicate that the transmission is restrained across the line defect due to the helical edge states flowing inversely along the pseudoedge

In two-dimensional (2D) materials such as graphene and transition metal dichalcogenides(MoS_2_, etc.), grain boundaries between two domains of material with different crystallographic orientations are ideal choices to achieve the valley polarization and has attracted considerable attention[9–14]. Recently, the extended line defects (ELDs) in silicene have been extensively investigated according to first-principles calculations[15, 16], and the 5-5-8 ELD (abbreviated as "line defect” in the following) was found to be the most stable and most readily formed structure. The spin and valley polarization of the silicene line defect have been investigated theoretically[17–19]. The formation of a line defect can be visualized as the stitching of the zigzag edges of two Si grains by the adsorbed Si atoms, where either side of the line defect shows pseudoedge-state-like behavior and the grain boundaries of the zigzag edge act as the pseudo-edge[16]. Obviously, such a lattice has mirror symmetry with respect to the line defect and the corresponding lattice vectors in the “left” and “right” domains separated by the defect are contrary[10, 11]. In such a line defect with inversion domain boundary, the *A*/*B* sublattices and valley indexes are exchanged upon crossing the defect. The line defect is semitransparent for the quasiparticles in graphene and a high valley polarization appears with a high angle of incidence. The valley polarization is *q*_*y*_ (the electron’s group velocity along the *y* direction) dependent across the line defect. For graphene, which has a linear dispersion and constant group velocity, the valley polarization can reach near 100% at large |*q*_*y*_| (corresponding to high angle of incidence) while it decreases as |*q*_*y*_| diminishes and vanishes as |*q*_*y*_|∼0 [9, 14]. In contrast, silicene has two different transmission characteristics [17, 18]: firstly, the two valleys become indistinguishable as the Fermi energy is close to the band edge due to the parabolic dispersion relation, and secondly, the transmission is restrained because of the helical edge state flowing inversely on both sides of the line defect, as shown in Fig. 1c. Naturally, the system with SOC in a particular RSOC is a promising candidate for efficient spin FET. The RSOC generates an in-plane effective magnetic field and induces the spin precession that is injected perpendicular to the plane of confinement. The spin polarization[20] and inversion[21] have been investigated in gated silicene nanoribbons. Theoretical calculations have shown that the energy band of silicene can be significantly modulated by RSOC [22, 23]. For instance, at a relative strong RSOC, the spin-down (-up) band at the *K*(*K*^′^) valley shifts up while the other spin bands in the conduction band remain unchanged. In consideration of the peculiar transmission feature in the silicene line defect and the effect of RSOC in silicene, the practical all-electric schemes for generating valley-polarized carriers becomes feasible.

In this paper, we propose an efficient way to polarize the Dirac fermions of different valleys using the silicene double line defects, thus creating distinct valley polarization by utilizing the electric field in silicene. Our results show that when the Fermi energy is near the bottom of conduction band, the oscillation images of the transmission coefficients from two valleys, which comprise wide oscillating peaks and nadirs, coincide as long as RSOC exceeds the intrinsic SOC, while the presence of only a single line defect cannot disperse the valley-dependent electrons. When two parallel line defects are involved, the oscillating nadirs evolve into zero-transmission plateaus, and effective modulation of valley-dependent transport can be realized by changing the oscillation periodicity of the two Dirac valleys with a perpendicular electric field, where the oscillation periodicity of the two valleys increases and decreases and leads to the perfect valley polarization at the wide peak-zero plateau corresponding regions. In experiment, one can detect such pure valley current by measuring the change of conductance with the electric field. This phenomenon provides a different route for effectively modulating the valley polarization in silicene devices by utilizing the RSOC and electric fields.

## Methods

Let us start from the schematic of a two-terminal silicene line defect device, as shown in Fig. 1a, in which the spin precession is illustrated to generate the valley-polarized current due to the RSOC and electric field. It is supposed that RSOC exists on one side of the line defect with width *W* and *WR* in units of $\sqrt {3}a$, where *a*=3.86 Å is the lattice constant of pristine silicene, as shown in Fig. 1a. When the Fermi energy is located at the bottom of conduction band, the states (*K*,*↓*)[ (*K*,*↓*) corresponds to a state in valley *K* with *↓*(down) spin] and (*K*^′^,*↑*) are in the gap due to the manipulation of energy band from RSOC. The other two states, (*K*,*↑*) and (*K*^′^,*↓*), circulate along the pseudo-edge because of the spin-momentum locking characteristic from SOC[24], as shown in Fig. 1a. For a definite spin state, it flows along the pseudo-edge with opposite directions on both sides of the line defect which can act as a filter and restrain the transmission across the line defect, as depicted in Fig. 1c.

A lattice model in the tight-binding representation is used to describe the line defect system with RSOC as [17, 22]

1 $$\begin{array}{@{}rcl@{}} H&=&t\sum_{\langle i,j\rangle\alpha}c_{i\alpha}^{\dag}c_{j\alpha}+\tau_{2}\sum_{\langle \gamma\delta\rangle\alpha}c_{i_{y}\alpha,\gamma}^{\dag}c_{i_{y}\alpha,\delta}+ \tau_{1}\sum_{\langle i,\gamma\rangle\alpha}c_{i\alpha}^{\dag}c_{i_{y}\alpha,\gamma}\\ &+&i\frac{t_{so}}{3\sqrt{3}}\sum_{\langle\langle i,j\rangle\rangle\alpha\beta}\nu_{ij}c^{\dag}_{i\alpha}\sigma_{\alpha\beta}^{z}c_{j\beta}+\Delta_{z}\sum_{i\alpha}\mu_{i} c_{i\alpha}^{\dag}c_{i\alpha}\\ &+&it_{R}\sum_{\langle i,j\rangle\alpha\beta}c_{i\alpha}^{\dag}(\vec{\sigma}\times\mathrm{\mathbf{d_{ij}}})^{z}_{\alpha\beta}c_{j\beta}+H.c., \end{array} $$

where $c_{i\alpha }^{\dag }$ and $c_{i_{y}\alpha,\gamma /\delta }^{\dag }$ represent the electron creation operator with spin *α* at silicene site *i* and the line defect, respectively, and 〈〉/〈〈〉〉 runs over all nearest-/next-nearest-neighbor-hopping sites. The first three terms denote nearest-neighbor hopping and the parameters *t*,*τ*_1_, and *τ*_2_ denote various nearest-neighbor hopping energies in the tight-binding model, as shown in Fig. 1b. The fourth term is the effective SOC with the hopping parameter *t*_*so*_, and *ν*_*ij*_=±1 for counterclockwise (clockwise) hopping between the next-nearest-neighboring sites with respect to the positive *z*-axis. A theoretical investigation [16] has shown that the two nearest Si atoms in the defect region are relatively identical to those in the pristine region and that all Si atoms remain in the *sp*^2^−*sp*^3^ hybridized state. Therefore, it is reasonable to set *τ*_2_=*τ*_1_=*t*. In the fifth term, *Δ*_*z*_ is the staggered sublattice potential that arises from an electric field perpendicular to the silicene sheet, and *μ*_*i*_=±1 for the *A*(*B*) site. The last term represents the extrinsic RSOC term where *t*_*R*_ is the Rashba spin-orbit hopping parameter. *d*_*ij*_ is the unit vector pointing from site *j* to *i*, and $\vec {\sigma }=(\sigma ^{x},\sigma ^{y},\sigma ^{z})$ in Eq. 1 is the vector of real spin Pauli matrices. The RSOC arises from external potential applied by either an electric gate, metal-atom adsorption or a substrates [20, 25] which can dramatically break the structure inversion symmetry of silicene. Notably, the extrinsic RSOC originating from the electric field is ignored because it is very weak.

The ELDs of silicene are shown in Fig. 1a, which extends immensely along the *y* direction. The translational symmetry of the lattice structure along the *y* direction indicates that *k*_*y*_ is a conserved quantity and that the creation (annihilation) operators can be rewritten as follows, according to the Fourier transformation (the spin index is ignored)[17]:

2 $$\begin{array}{@{}rcl@{}} c_{i}^{\dag}=\sum_{k_{y}}c_{k_{y},i_{x}}e^{-2ik_{y}i_{y}a},c_{i}=\sum_{k_{y}}c_{k_{y},i_{x}}e^{2ik_{y}i_{y}a},  \\ c_{i_{y},\gamma}^{\dag}=\sum_{k_{y},\gamma}c^{\dag}_{k_{y},\gamma}e^{-2ik_{y}i_{y}a},c_{i_{y},\gamma}=\sum_{k_{y},\gamma}c_{k_{y},\gamma}e^{2ik_{y}i_{y}a}. \end{array} $$

Then, the Hamiltonian matrix in Eq. 1 is decoupled into $H=\sum _{k_{y}}H_{k_{y}}$, where $H_{k_{y}}$ can be described in the following form:

3 $$ {\begin{aligned} H_{k_{y}}=-\sum_{i}\varphi_{i,1}^{\dag}\hat{T}_{11}\varphi_{i,1}-\sum_{i}\varphi_{i,2}^{\dag}\hat{T}_{22}\varphi_{i,2}\\ -\sum_{i}\varphi_{i,1}^{\dag}\hat{T}_{12}\varphi_{i,2}-\sum_{i\neq-1}\varphi_{i,2}^{\dag}\hat{T}_{23}\varphi_{i+\hat{x},1}\\ -\varphi_{\bar{1},2}^{\dag}\hat{T}_{\bar{1}0}\varphi_{0}-\varphi_{0}^{\dag}\hat{T}_{01}\varphi_{1,1}-\varphi_{0}^{\dag}\hat{T}_{00}\varphi_{0} -\varphi_{\bar{1},2}^{\dag}\hat{T}_{\bar{1}1}\varphi_{1,1}+h.c., \end{aligned}}  $$

where $\varphi _{i,l}^{\dag }=\left [ c_{{{k}_{y}},i,l,A\uparrow }^{\dag }, c_{{{k}_{y}},i,l,A\downarrow }^{\dag }, c_{{{k}_{y}},i,l,B\uparrow }^{\dag },c_{{{k}_{y}},i,l,B\downarrow }^{\dag }\right ]$, *i* in the set of index (*i,l*) represents the position of a supercell $(\bar {i}=-i)$, and *l*=1 or 2 denotes different zigzag chains in a supercell, as shown in the dashed rectangle in Fig. 1b. $\hat {T_{ll'}}$ represents the Hamiltonian matrix of each zigzag chain (*l*=*l*^′^) in a supercell or the interplay between different zigzag chains (*l*≠*l*^′^).

It is noted that the two valleys *K* and *K*^′^ are now cast at [0,±*π*/3*a*] due to the insertion of the line defect. The transmission matrix of the *η*(*η*=*K*/*K*^′^) valley is calculated using the generalized Landauer formula[26, 27],

4 $$\begin{array}{@{}rcl@{}} T={\left(\begin{array}{cc} T^{\uparrow\uparrow}_{\eta} & T^{\uparrow\downarrow}_{\eta} \\ T^{\downarrow\uparrow}_{\eta} & T^{\downarrow\downarrow}_{\eta} \end{array} \right)}=\sum_{i,j=1}^{8}{ \left(\begin{array}{cc} \vert t_{ij,\eta}^{\uparrow\uparrow}\vert^{2} &\vert t_{ij,\eta}^{\uparrow\downarrow}\vert^{2} \\ \vert t_{ij,\eta}^{\downarrow\uparrow}\vert^{2} &\vert t_{ij,\eta}^{\downarrow\downarrow}\vert^{2} \end{array} \right)}, \end{array} $$

where

5 $$\begin{array}{@{}rcl@{}} t=2\sqrt{-Im\Sigma_{L}}G^{r}\sqrt{-Im\Sigma_{R}} \end{array} $$

and

6 $$\begin{array}{@{}rcl@{}} t_{ij,\eta}^{\uparrow\uparrow}&=t_{2(i-1)+1,2(j-1)+1}\\ t_{ij,\eta}^{\uparrow\downarrow}&=t_{2(i-1)+1,2j}\\ t_{ij,\eta}^{\downarrow\uparrow}&=t_{2i,2(j-1)+1}\\ t_{ij,\eta}^{\downarrow\downarrow}&=t_{2i,2j}. \end{array} $$

Here, $-Im\Sigma _{L,R}=-\left (\Sigma _{L,R}^{r}-\Sigma _{L,R}^{a}\right)/ 2i$ are positive semidefinite matrices with a well-defined matrix square root, where $\Sigma _{L,R}^{a}=\left [\Sigma _{L,R}^{r}\right ]^{\dag }$ are the retarded/advanced self-energy of the left/right lead. The 16×16 submatrix *G*^*r*^ is the retarded Green’s function, which connects the first and last supercells along the *x* direction and can be calculated using the recursive Green’s function method. The total transmission coefficients of the *η* valley are $T_{\eta }=T^{\uparrow \uparrow }_{\eta }+T^{\uparrow \downarrow }_{\eta }+ T^{\downarrow \uparrow }_{\eta }+T^{\downarrow \downarrow }_{\eta }$, and the spin polarization *P*_*s*_ and valley polarization *P*_*η*_ can be given by

$${\begin{aligned} P_{s}&=\frac{T_{K}^{\uparrow\uparrow}+T_{K}^{\uparrow\downarrow}-T_{K}^{\downarrow\downarrow}-T_{K}^{\downarrow\uparrow}+T_{K'}^{\uparrow\uparrow}+T_{K'}^{\uparrow\downarrow}-T_{K'}^{\downarrow\downarrow}-T_{K'}^{\downarrow\uparrow}}{T_{K}+T_{K^{\prime}}},\\ P_{\eta}&=\frac{T_{K}-T_{K^{\prime}}}{T_{K}+T_{K^{\prime}}}. \end{aligned}} $$

## Results and Discussion

In the calculations of the spin-dependent transmission coefficients, we set *τ*_2_=*τ*_1_=*t*=1 as the energy unit, the SOC strength *t*_*so*_=0.005*t*, and the Fermi energy *E*_*f*_=1.001*t*_*so*_, which is situated at the bottom of the conduction band. The width of the scattering region is *W*=1000 for the single line defect and an additional width WR=1000 is also taken into account for the two parallel line defects, as shown in Fig. 1a.

**Fig. 2:**
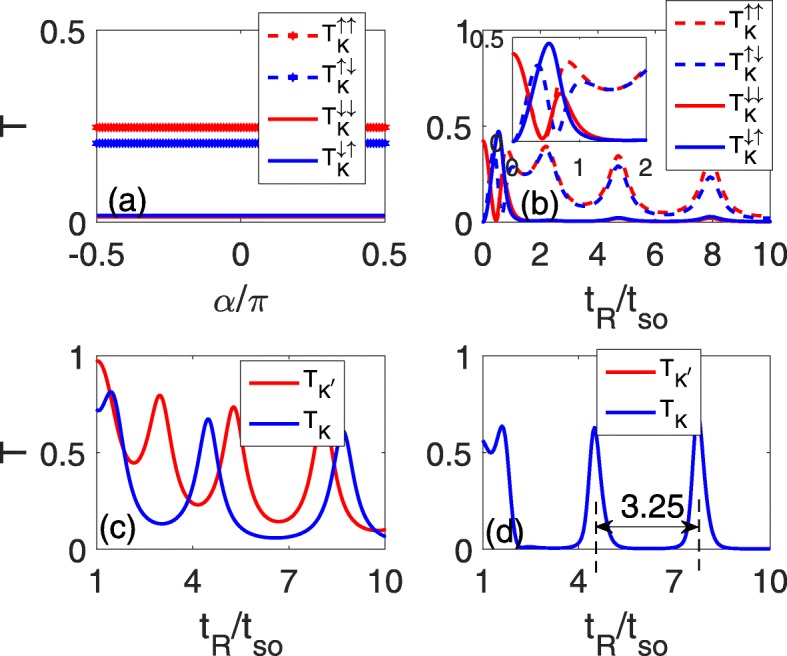
Spin-conserved and spin-flip transmission coefficients as functions of the incident angles *α* at *t*_*R*_=5*t*_*so*_ in **a** and as functions of the RSOC strength *t*_*R*_ in **b**–**d**, where **a**-**c** are for the single line defect and **d** is for the two parallel line defects, with *Δ*_*z*_=0.2*t*_*so*_ in **c**

Figure 2 depicts the spin-conserved/spin-flip transmission coefficients of valley $\eta, T^{sc}_{\eta }/T^{sf}_{\eta }$, as a function of the incident angles *α* (a) and of the RSOC strength *t*_*R*_ (b–d). Figure 2a–c correspond to the case of the single line defect, and (d) is for the case of the two parallel line defects. It is shown that at a definite *t*_*R*_ (for instance, *t*_*R*_=5*t*_*so*_ as in Fig. 2a), the spin-dependent transmission coefficients $T^{sc}_{K}/T^{sf}_{K}$ are constant and independent of the incident angles due to the parabolic dispersion relation, as shown in Fig. 2a. Therefore, in the following calculations, we can use the incident angle *α*=0 as an example. For a weak *t*_*R*_, an oscillating phenomenon similar to that in a two-dimensional electron gas [26, 27] appears due to the Rashba splitting, as shown in the inset of Fig. 2b. As *t*_*R*_ increases (*t*_*R*_>*t*_*so*_), $T_{K}^{\uparrow \uparrow }$ and $T_{K}^{\uparrow \downarrow }$ have the same oscillating periodicity and nearly the same magnitudes as *t*_*R*_ which consists of some oscillation peaks and nadirs, while $T_{K}^{\downarrow \downarrow }/T_{K}^{\downarrow \uparrow }$ tends to zero because the Fermi energy lies in its gap, as shown in Fig. 2b. Thus, the total transmission coefficient of *K* valley is mainly contributed by the spin up state. In fact, the oscillation images of the two valleys, *K* and *K*^′^, coincide while the transmission coefficients of *K*^′^ valley is mainly contributed by the spin-down electrons.

In the presence of a perpendicular electric field, the valley degeneracy is lifted, and the oscillating behaviors of the two valleys differs: the oscillating periodicity of the *K* valley increases, while that of the *K*^′^ valley decreases, as shown in Fig. 2c. However, it seems infeasible to filter one conical valley state with only a single line defect because the oscillating nadirs have a definite magnitude. Naturally, one may consider the oscillating phenomenon with two parallel line defects to further restrain the transmission, as shown in Fig. 2d. Comparing Fig. 2b with d reveals that the oscillation peak becomes narrow and acute, while the oscillation nadir broadens and weakens, which forms the zero-transmission platform. The space between two neighboring oscillation peaks is fixed at 3.25*t*_*so*_, as characterized by the two dashed lines in Fig. 2d.

**Fig. 3:**
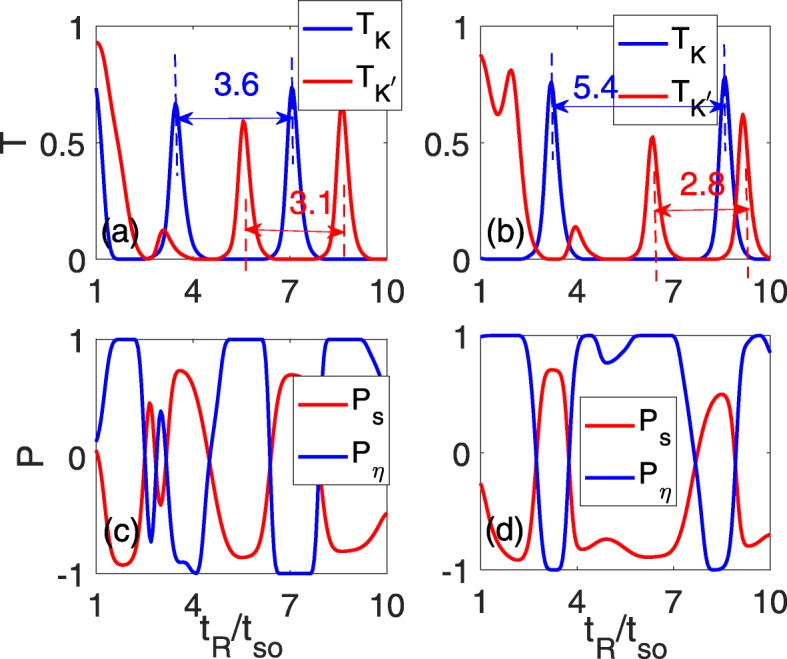
The total transmission coefficients $T_{K}/T_{K^{\prime }}$ (**a**, **b**) and the spin/valley polarization (**c**, **d**) as a function of the RSOC strength *t*_*R*_ for different sublattice potentials. *Δ*_*z*_=0.15*t*_*so*_ in **a** and **c** and *Δ*_*z*_=0.3*t*_*so*_ in **b** and **d**; the other parameters are identical to those in Fig. 2d

To achieve a better valley filter effect, we concentrate our attention on the effect of the perpendicular electric field. The results of this effect are shown in Fig. 3. As discussed above, the oscillating periodicity of the two valleys change in an opposite manner, and the original overlapping oscillation peaks in Fig. 2d are relieved. Meanwhile, the zero-transmission plateau broadens and narrows for *T*_*K*_ and $T_{K^{\prime }}$, respectively, as shown in Fig. 3a and b. At *Δ*_*z*_=0.15*t*_*so*_, the space between the two neighboring oscillation peaks develops into 3.6*t*_*so*_ for *T*_*K*_, while it is reduced to 3.1*t*_*so*_ for $T_{K^{\prime }}$, as indicated by the two blue and red dashed lines shown in Fig. 3a. As the electric field strengthens, the space between the two neighboring oscillation peaks continues to increase/decrease for *T*_*K*_/$T_{K^{\prime }}$, which is 5.4*t*_*so*_/2.8*t*_*so*_ at *Δ*_*z*_=0.3*t*_*so*_, as shown in Fig. 3b. The change in the oscillation periodicity will lead to the corresponding regions of wide peak-zero plateau, where perfect valley polarization with *P*_*η*_=±1 plateaus can be realized, as shown in Fig. 3c and d. Simultaneously, it is shown that high spin polarization *P*_*s*_ also arises when *P*_*η*_=±1.

**Fig. 4:**
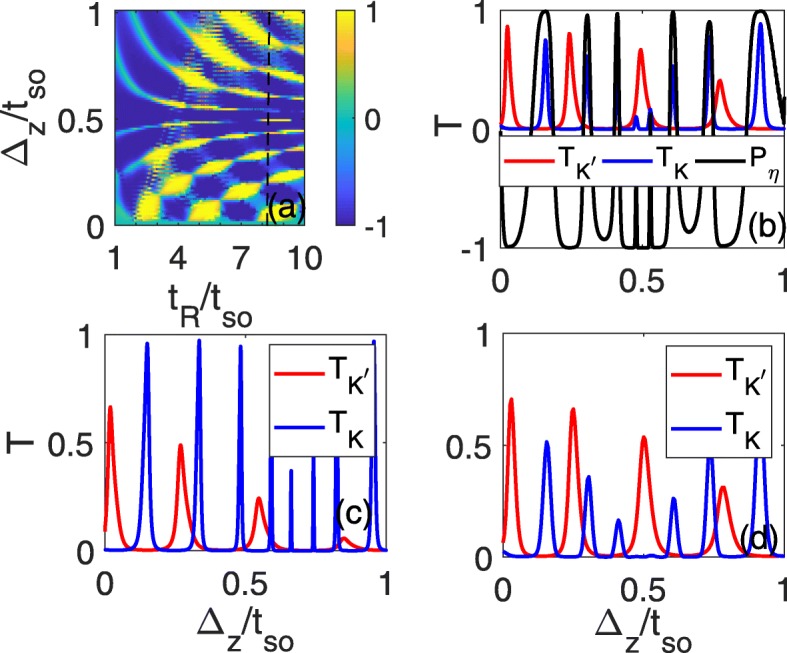
Valley polarization **a** and $T_{K}/T_{K^{\prime }}$ (**b**–**d**) as functions of *Δ*_*z*_ and *t*_*R*_. *t*_*R*_=7.2*t*_*so*_ in (**b**–**d**), *E*_*f*_=1.5*t*_*so*_ in **c** and **d**, and the on-site energy is *E*=0.15*t* in the left electrode in **d**; all other parameters are identical to those in Fig. 2d

However, due to the uncontrollability of RSOC, it is still difficult to detect such pure valley currents experimentally, even though the RSOC induced in the line defect can be greater than the intrinsic SOC. To conveniently probe the pure valley current experimentally, we also investigate the transmission coefficients and valley polarization as a function of electric field, which can be continuously controlled during an experiment. It is shown that the perfect valley polarization with *P*_*η*_=±1 can emerge in a certain range of *Δ*_*z*_ and that it can change from *P*_*η*_=1 to *P*_*η*_=−1 as the electric field increases, as shown in Fig. 4a. For a definite *t*_*R*_ (for instance *t*_*R*_=7.2*t*_*so*_, as indicated with a dashed line in Fig. 4a), the transmission coefficients $T_{K}/T_{K^{\prime }}$ oscillate with *Δ*_*z*_, where the wide transmission peaks of the *K*(*K*^′^) valley correspond to the zero-transmission plateaus of the *K*^′^(*K*) valley. The total transmission coefficients are basically contributed by one valley as the electric field varies, and perfect valley polarization can always occur around the maximal value of $T_{K}/T_{K^{\prime }}$, as shown in Fig. 4b. As the Fermi energy departs from the band edge, the perfect valley polarization can still survive even at *E*_*f*_=1.5*t*_*so*_, where the plateau relation can be well maintained, as shown in Fig. 4c. During an experiment, one can analyze the valley-polarized electrical currents from the left to right lead with an experimentally measurable quantity such as the conductance, which is proportional to the total transmission coefficient. The maximal conductance between two minimum values (sometimes, they are zero) should be from one valley. We can estimate the magnitude of the conductance according to the formula $G=\frac {e^{2}}{h}\int _{-k_{F}}^{k_{F}}T\frac {dk_{y}}{2\pi /L_{y}}=\frac {e^{2}}{h}\frac {Ly\sqrt {E^{2}-t^{2}_{so}}}{2\pi \hbar v_{F}}2T$ [28], where *L*_*y*_=2*a*≈7.72Å is the width of silicene line defect, *v*_*F*_=5.5×10^5^*m*/*s* is the Fermi velocity, $\hbar =h/2\pi $ is the reduced Planck constant with $\phantom {\dot {i}\!}h=4.13566743\times 10^{-15}eV\cdot s, T=T_{K}+T_{K'}$ is the total transmission coefficient and *E* is the on-site energy of the incident electrons. Then, the conductance is about $G\approx \left [0.7T\sqrt {E^{2}-t^{2}_{so}}/eV\right ]\frac {e^{2}}{h}$. It is also found that as the on-site energy in the incident side is raised to *E*=0.15*t*(*t*=1.6*eV*), the transmission coefficients of the two valleys change only a little compared with Fig. 4c due to spin and momentum conservation and the transmission peak-zero plateau relation maintains still, as shown in Fig. 4d. In this case, the conductance is about $G\approx 0.17T\frac {e^{2}}{h}$ which is sizable and can be detectable in experiment. The energy window to observe this phenomenon is about 0.5*t*_*so*_(*t*_*so*_<*E*<1.5*t*_*so*_) which is proportional to *t*_*so*_. In experiment, it is not difficult to control the Fermi energy near the band edge and the SOC gap can even be radically increased to 44 meV by proximity with Bi(111) bilayer[29] which can greatly improve the energy region to detect the pure valley current. Moreover, the computational model can also be applicable to other low-buckled counterparts of graphene, germanene[30],stanene and MoS_2_[31–36],which have even larger band gaps[37, 38] as well as the SOC strengths(SOC strength can reach 0.1eV for stanene[38, 39]). In a real experiment, it is easy to realize a strong RSOC which can exceed the intrinsic SOC by breaking the in-plane mirror symmetry with the special substrate[40]. Therefore, this scheme can be completely feasible in experiment.

## Conclusions

We have proposed an electrical method for generating a valley-polarized current in silicene line defects. In sharp contrast to the conventional electrical approaches that are used to produce valley-polarized current, we explore the RSOC, which is considered to tune the widely used spin polarization in spin-polarized FETs. It is found that the transmission coefficients of the two valleys oscillate with the same periodicity and intensity, which is composed of transmission peaks and zero-transmission plateaus. The valley-polarized current can be generated by tuning the oscillating periodicity of the two valleys with an electric field, which can destroy the symmetry of the valley states and bring about the corresponding transmission peak-zero plateau regions. Moreover, we also provide a scheme to detect the pure valley current in experiment and the results may shed light on the manipulation of valley-polarized currents by electrical means.

## Data Availability

The datasets generated during and/or analyzed during the current study are available from the corresponding authors on reasonable request.

## Abbreviations

2D: Two-dimensional
ELD: Extended line defect
FET: Field-effect transistor
RSOC: Rashba spin-orbit coupling
SOC: Intrinsic spin-orbit coupling
